# Phenotypic and Comparative Transcriptome Analysis of Different Ploidy Plants in *Dendrocalamus latiflorus* Munro

**DOI:** 10.3389/fpls.2017.01371

**Published:** 2017-08-08

**Authors:** Guirong Qiao, Mingying Liu, Kunlin Song, Haiying Li, Huiqin Yang, Yafang Yin, Renying Zhuo

**Affiliations:** ^1^State Key Laboratory of Tree Genetics and Breeding, Chinese Academy of Forestry Beijing, China; ^2^Key Laboratory of Tree Breeding of Zhejiang Province, The Research Institute of Subtropical Forestry, Chinese Academy of Forestry Hangzhou, Zhejiang, China; ^3^Wood Anatomy and Utilization Department, Research Institute of Wood Industry, Chinese Academy of Forestry Beijing, China

**Keywords:** Ma bamboo, digital gene expression, ploidy, coexpression network, growth properties, anatomical characteristics

## Abstract

Elucidating the differences in gene expression profiles of plants with different ploidy levels and how they affect phenotypic traits is vital to allow genetic improvement of plants such as Ma bamboo (*Dendrocalamus latiflorus* Munro). We previously obtained triploid (2n = 3X = 36), hexaploid (2n = 6X = 72), and dodecaploid (2n = 12X = 144) Ma bamboo plants from embryogenic callus by anther culturing. Phenotypic differences between these plants appeared to be correlated with differences in ploidy. Here, we performed transcriptome profiling and sequencing of anther-regenerated plants and F1 seedlings of different ploidy levels using RNA-Seq technology. Pair-wise comparisons of the four resulting libraries revealed 8,396 differentially expressed genes. These differentially expressed genes were annotated, functionally classified, and partially validated. We found that the chromosome doubling led to substantially up- or down-regulation of genes that were involved in cell growth and differentiation; the polyploidy levels altered the anatomical, physiological and growth characteristics, such as leaf thickness, fusoid cell and stomatal size, shoot number, photosynthesis and respiration rate and so on. Additionally, two candidate genes, *EXPB3* and *TCP* with potenitial regulatory roles in cell division and differentiation, were identified through gene coexpresseion network analysis. These results highlight the significance of potential applications of polyploidy, and provide valuable information for the genetic breeding of bamboo species.

## Introduction

The group of bamboo species, which are perennial woody plants and belongs to the grass family (Poaceae, Bambusoideae, Bambuseae), has considerable economic and cultural significance (Group et al., 2001). Bamboo is an important forest resource due to its fast growth, excellent specific strength, and easy processing (Chang and Wu, 2000). Bamboo is a particularly significant component of tropical and subtropical forest ecosystems. It has a long history of use as a forest product for furniture, paper pulp, and construction materials and as a garden ornamental plant. Ma bamboo (*Dendrocalamus latiflorus* Munro) is large tropical clumping bamboo species native to southern China. Its young shoots are edible. The culms can grow up to about 20 m in height and 20 cm in diameter, whereas the mature culms can be used for construction and paper-making.

Genetic improvement using traditional cross breeding is difficult for most bamboo species, due to the long juvenile stage and low seed setting. As an alternative breeding method, anther culture has been used wildly in the grass family including important cereal crops, such as wheat, barley (Zivy et al., 1992), and rice (Zhuo et al., 1995). Chromosome doubling is easy during anther culture of haploid cells and tissues (Zhuo et al., 1995). Compared to diploids, genome doubling in polyploids confers distinct advantages in growth, natural selection and reproduction which allow polyploids to thrive in various environments (Madlung, 2013). Although the reasons of novel variation in polyploids are not well understood, they might involve changes in gene expression through dosage-regulated gene expression, altered regulatory interactions, and rapid genetic and epigenetic changes (Osborn et al., 2003). Dosage-regulated gene expression occurs when the level of gene expression is positively correlated with gene copy number. In general, polyploidy leads to increased gene expression levels on a per cell basis in proportion to the gene dosage conferred by the ploidy level, as was shown for most genes in a euploid series (monoploid, diploid, triploid, and tetraploid) of maize (Guo et al., 1996). Genes duplicated by polyploidy may retain their original or similar functions, or they may undergo diversification in protein function or regulation. Alternatively, one gene copy may become silenced through mutational or epigenetic means (Wendel, 2000).

To address this question, high-throughput transcriptome sequencing and digital gene expression (DGE) tag profiling, as efficient and cost-effective techniques were used for characterizing non-model organisms without a reference genome (Wang et al., 2009; Surget-Groba and Montoya-Burgos, 2010). We previously established an efficient plant-regeneration system for *D. latiflorus* by anther culture and obtained triploid (2n = 3X = 36), hexaploid (2n = 6X = 72), and dodecaploid (2n = 12X = 144) plants (Qiao et al., 2013). Among the bulk of the regenerated bamboo plants, which are homozygotes, we observed that the plantlets exhibit different phenotypes such as differences in chlorophyll content (Qiao et al., 2013). We also took advantage of the availability of a Ma bamboo flower transcriptome (Ma et al., 2012) and transcriptome data for Ma bamboo from roots, stems, flowers, seeds, and shoots (Liu et al., 2012).

In this study, we characterized the differences in morphological and physiological characteristics of Ma bamboo at different ploidy levels. To investigate potentially related differences in gene expression patterns and how these differences affect phenotypic traits in plants with different ploidy, we compared the gene expression profiles of four plants with different ploidy levels and constructed a coexpression network of differentially expressed genes. The results of this study help elucidate the molecular mechanisms underlying bamboo growth and development, and they provide a valuable resource for future genetic and genomic studies of Ma bamboo.

## Materials and methods

### Plant materials

Three homozygotes generated from anthers exhibiting different ploidy levels were used in comparison with seedlings cultured from seeds. For DGE sequencing analysis, the triploid line showing retarded growth was designated as Munro_3, the dodecaploid was designated as Munro_12, the hexaploid line was designated as Munro_6, and the seedlings used as the control were designated as Munro_10. Three biological repeats were performed per libraries.

### Optical microscopy

The third true leaves were selected from the first branch of the bamboo stem, and the middle parts of the leaves were immersed into formaldehyde, acetate, and alcohol (FAA) fixative. Small pieces cut from the midrib and intercostal regions of bamboo leaves at median level were dehydrated through a graded ethanol series and embedded in Spurr's resin (Spurr, 1969). Transverse and longitudinal Sections Conclusion μm in thickness were cut with an ultramicrotome (LKB 2188) and stained with a 1% aqueous solution of toluidine blue. Morphology was observed under a light microscope (Olympus BX61). Images were recorded using the Charge-coupled device (CCD) attached to the microscope and analyzed with image processing software (CorelDraw X5). The mean value was averaged from the data from at least 20 individual cells per ploidy level. Stomatal size and density were measured by the silicone rubber impression technique (Weyers and Johansen, 1985). Twenty stomata per genotype lengthwise were measured for stomatal size. the number of stomata per square millimeter was calculated for stomatal density.

### Growth and physiological characteristics

Growth properties include plant height, stem diameter, and shoot number were measured and calculated for three periods of shoot development (May to June, July to August, and September to October). Photosynthesis and transpiration rates were examined with an Li-6400 XT Portable Photosynthesis System with an Artificial light source (PAR, 800 μmol m^−2^ s^−1^; Flow, 500 μmol s^−1^) in the greenhouse at 9:00–11:00 a.m. during the period of rapid growth of Ma bamboo.

Three biological repeats were performed per index. There are no data about the growth and physiological characteristics of the triploid lines due to their lack of vigor. Statistical analysis was carried out to quantitate the differences in characteristics at different ploidy levels using SPSS software version 15. The differences between the means were scored by Duncan's multiple range test (Duncan, 1955).

### RNA-Seq

#### RNA extraction

Roots, stems, and leaves were dissected, immediately snap-frozen in liquid nitrogen, and stored at −80°C until further processing. Total RNA was extracted using Total RNA purification kit (Norgen Bioket, Canada) and treated with DNase I (Invitrogen, USA) according to the manufacturer's instructions. RNA quality was quantified assessed based on OD260/280 ratio using a ND-2000 spectrophotometer (NanoDrop Technologies, Wilmington, DE, USA). RNA purity were tested by an Agilent 2100 Bioanalyzer (Agilent Technologies, Santa Clara, CA, USA) based on RNA integrity number (RIN). The RNA of the three tissues was mixed at equal proportions.

#### Preparation of sequencing libraries

Sequencing libraries were prepared following the manufacturer's instructions of Illumina Gene Expression Prep kit (Illumina Inc., San Diego, CA, USA). In briefly, the poly-(A) mRNA was purified using oligo (dT) magnetic beads and then fragmented by the RNA fragmentation kit (Ambion, Austin, TX). Double-stranded cDNA was synthesized by SuperScript II Reverse Transcriptase (Invitrogen, Carlsbad, CA, USA) and end-repaired, followed by a single A. Adapters for sequencing were ligated to cDNA fragments using T4 DNA ligase (NEB). Adaptor-ligated cDNA fragments were amplified by PCR. The PCR products were separated purified on an agarose gel. 150–200 bp PCR products were excised from the gel and retrieved using a Gel Extraction Kit (Axygen Biosciences, Central Avenue Union City, CA) purified by ethanol (Illumina Inc.). Quality control was performed to quantify the DNA concentration and validate the library. Purified libraries were diluted to a 10 nM concentration and stored at −20°C until sequencing.

#### Sequencing and annotation

Each DGE library was sequenced by a single end read protocol. Data collected per run on the Illumina Genome Analyzer IIx sequencing platform. Image analysis and base calling were performed by the Illumina instrument software. Adapter sequences were removed from the raw reads. Low-quality sequences (Q < 20 bases) were discarded. Clean reads were counted by using custom Perl scripts written for DGE analysis. DGE tags were annotated to the Ma bamboo transcriptome, which was obtained through our former work (Liu et al., 2012). Tag counts were normalized by Tags per million (TPM) for each library. Total expression for each gene was calculated by summing all tags mapped to the same gene.

### Analysis of differentially expressed genes

To compare the differential gene expression patterns among the four libraries, the tag distribution for gene expression level in each library was normalized to obtain an effective library size. Significant differentially expressed transcripts (DETs) with *p* < 0.05 and log2 fold-change > 1 were extracted using edgeR (Empirical analysis of Digital Gene Expression in R) (Robinson et al., 2010).

Transcript levels were quantified in reads per kilobase of exon model per million mapped reads (RPKM) (Mortazavi et al., 2008). The DETs were blast in the Swiss-prot database, NCBI Non-redundant (Nr) database and Nt database. The differentially expressed sequences were assigned to GO terms describing biological processes, molecular functions and cellular components by Blast2GO (Conesa et al., 2005; Conesa and Götz, 2008). The data represented a GO analysis at level 2, illustrating general functional categories.

Gene functions of the differentially expressed sequences were predicted and classified according to the COG database. KEGG pathways were also analyzed using the online KEGG Automatic Annotation Server (KAAS), http://www.genome.jp/kegg/kaas/. The results of KEGG analysis contained KO assignments and KEGG pathways. Heatmaps of gene expression were generated using Heatmap Illustrator, version 1.0.

### Quantitative RT-PCR

The expression levels of 11 genes identified by DGE were validated by quantitative RT-qPCR performed on a 7300 Real Time PCR (Applied Biosystems, Carlsbad, CA, USA). The RNA samples were same as for the sequencing mentioned above. The cDNA was synthesized from 500 ng of total RNA using a PrimeScript 1st Strand cDNA Synthesis kit (TakaRa Bio, Dalian, China). *Glyceraldehyde-3-phosphate dehydrogenase* (*GAPDH*) was used as an endogenous control, as it was the housekeeping gene (Liu et al., 2012). The primers for RT-qPCR were designed using the Primer5 software (Table S1). RT-qPCR was performed using SYBR® Premix Ex Taq™ Kit (TakaRa Bio, Dalian, China). Each cDNA samples were analyzed in triplicate The 2^ΔΔ^Ct method was used to calculate relative expression levels (Livak and Schmittgen, 2001; Moriya et al., 2007).

### Co-expression network construction

Network construction was performed to identify modules of highly correlated genes by weighted correlation network analysis (WGCNA) (Zhang and Horvath, 2005; Langfelder and Horvath, 2008). Expression values transformed to log2 values based on the DGE data. Quality control were analysised by clustering and principal component analysis according to the gene expression levels in the DGE data set. The correlation networks were performed by the WGCNA package (version 1.46) in R (version 3.1.3) (Venables et al., 2012). Eigengenes were calculated to visualize gene expression patterns for each gene coexpression module. The co-expressed genes with strong interconnections were defined as hub genes. Subsequently, differentially expressed genes were selected from the hub genes and their edges were analyzed. The results of gene co-expression analysis were visualized by Cytoscape software (version 3.2.1) (Saito et al., 2012).

## Results

### Anatomical characteristics of Ma bamboo leaves of different ploidy

**Figure 1:**
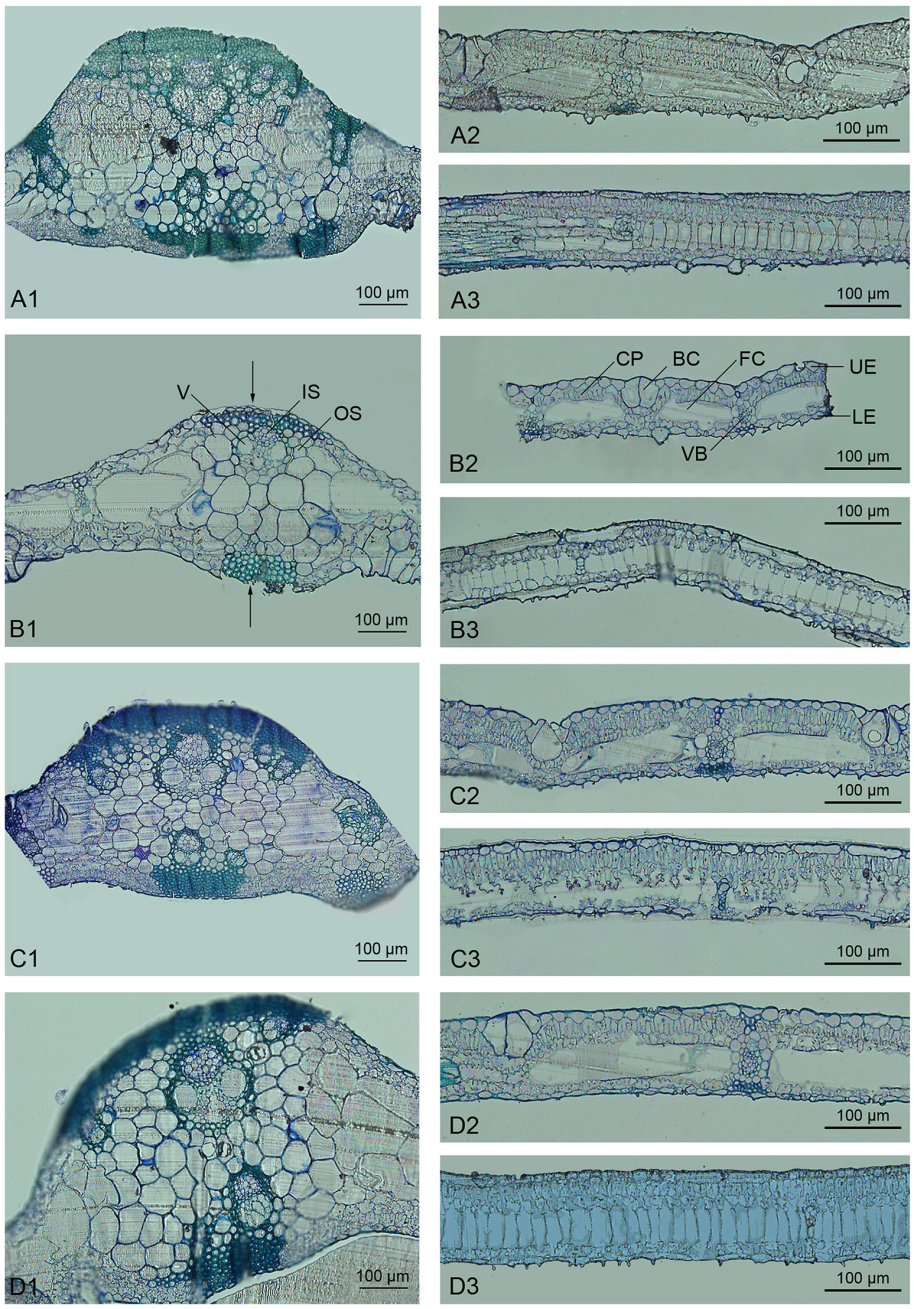
Light micrographs of the leaves of *Dendrocalamus latiflorus* plants of different ploidy levels. V, Vessel; IS, Inner sheath; OS, Outer sheath; CP, Chlorophyllous parenchyma; BC, Bulliform cell; FC, Fusoid cell; UE, Upper epidermis; LE, Lower epidermis; VB, Vascular bundle. **(A1–A3)**, F1 seedlings 6X; **(B1–B3)**, triploid 3X; **(C1–C3)**, hexaploid 6X; **(D1–D3)**, dodecaploid 12X; **(A1,B1,C1,D1)**, transverse sections of midrib; **(A2,B2,C2,D2)**, intercostal regions; **(A3,B3,C3,D3)**, longitudinal section of intercostal regions, arrow indicates the sheath extension.

Bamboo leaves commonly consist of the epidermis, chlorophyll, and vascular tissue. As confirmed by microscopic analysis, the epidermis in *Dendrocalamus latiflorus* Munro is composed of both long and short cells; the former contain sinuous walls, while the latter often occur in pairs or in isolation, with non-sinuous walls (Soderstrom and Ellis, 1988). Here we found that plants of all ploidies examined showed similar microstructure (Figure 1). Bulliform cells, which are an intrinsic part of the epidermis that allow the leaf to bend or fold inward to avoid excess water loss, were regularly distributed (e.g., Figure 1B2). The vascular system in the bamboo leaf was oriented in the longitudinal direction, with collateral vascular bundles of various sizes enclosed in a double sheath. The outer sheath consisted of parenchymatous cells, and the inner sheath comprised sclerenchymatous pericycle formed by cells with small dimensions and thickened walls (e.g., Figure 1B1). Sheath extension of a sclerenchymatous nature could be observed in the vascular bundles of bamboo leaves (e.g., Figure 1B2). The midrib of the bamboo leaf was distinct and contained several vascular bundles. The remaining space between the vascular tissue and epidermis was mainly filled with two types of thin-walled chlorophyll cells, i.e., fusoid cells and chlorophyllous parenchyma (e.g., Figure 1B2). Fusoid cells were found on both sides of the vascular tissue in perpendicular orientation. These cells likely function in the storage and transport of water in the mesophyll, considering their location next to vascular bundles (Vieira et al., 2002). Chlorophyllous parenchyma with vertically orientated invaginations filled the spaces between the epidermis and fusoid cells.

**Figure 2:**
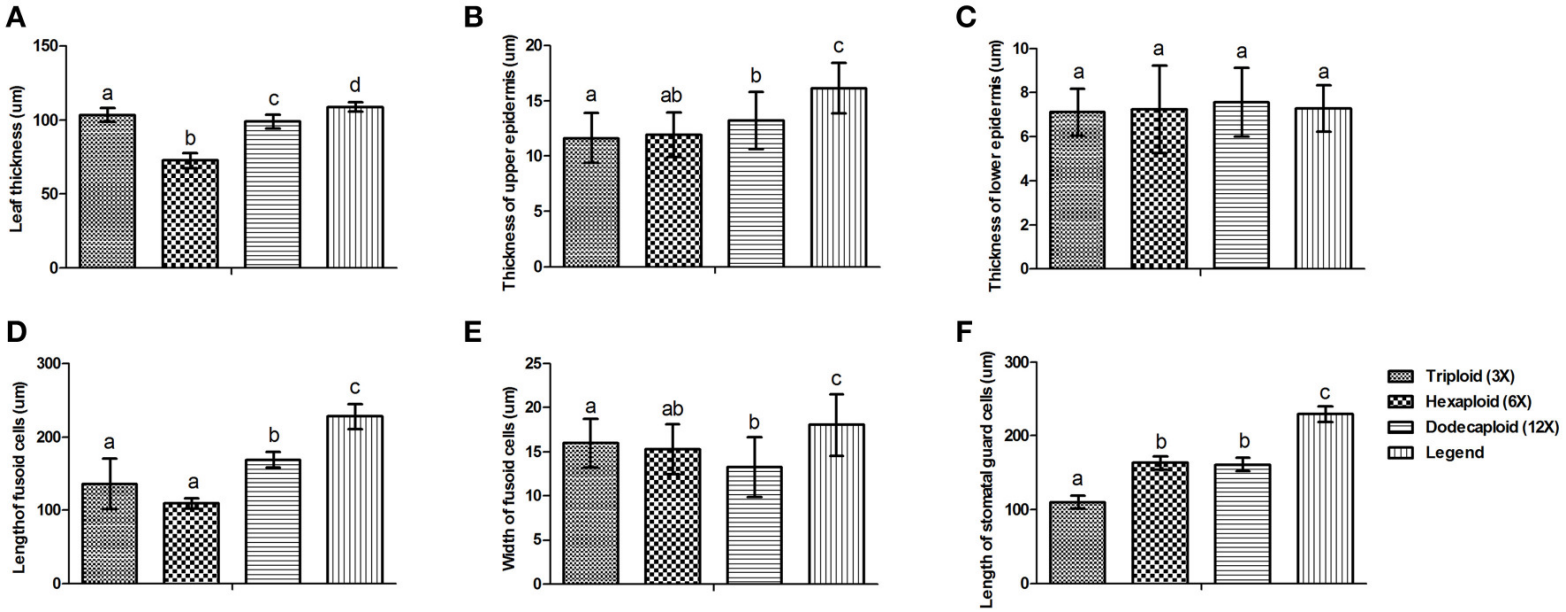
Differences in anatomical features of Dendrocalamus latiflorus plants of different ploidy levels. **(A)** Leaf thickness, **(B)** Thickness of upper epidermis, **(C)** Thickness of lower epidermis, **(D)** Length of fusoid cells, **(E)** Width of fusoid cells, **(F)** Length of somatal guard cells. Different capital letters indicate a significant difference between ploidy levels at *p* < 0.05 (Duncan, 1955).

There were significant variations in the anatomical features of bamboo leaves from plants with different ploidy levels (Figure 2). The average thickness of the dodecaploid bamboo leaves was significantly greater than that of the other leaves, whereas the triploid had the thinnest leaves (Figure 2A). The dodecaploid bamboo leaves had the thickest upper epidermis, whereas that of triploid leaves and seedlings was the thinnest (Figure 2B). No obvious difference in the thickness of the lower epidermis was observed in this study (Figure 2C). The dodecaploid bamboo also showed the biggest fusoid cells (Figures 2D,E). The stomatal size increased from triploids to hexaploids to dodecaploids (Figure 2F), whereas there was no obvious difference in stomatal density between lines.

### Growth and physiological characteristics vary with ploidy in Ma bamboo

**Figure 3:**
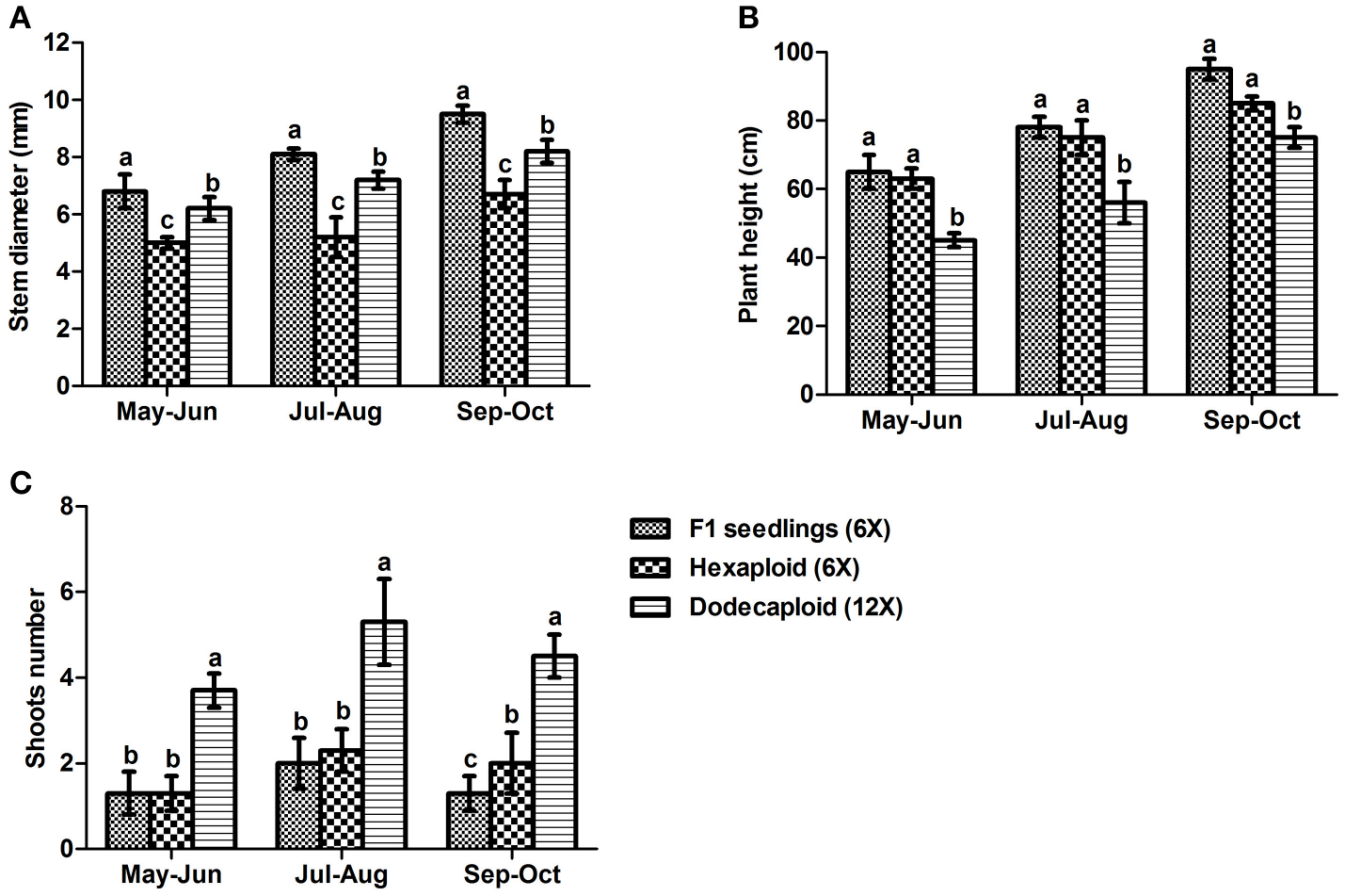
Comparison of growth characteristics of *Dendrocalamus latiflorus* lines of different ploidy levels. **(A)** stem diameter, **(B)** plant height, and **(C)** shoot number. Values followed by the same letter are not significantly different according to the least significant difference at *P* < 0.05 (Duncan, 1955).

We measured plant growth properties including plant height, stem diameter, and shoot number continuously from May to October 2013, finding that there were significant differences between seedlings and plants generated from anthers. Although the hexaploid and dodecaploid plants generated from anthers were shorter and had smaller stem diameters than seedlings, they tended to have more shoots. In particular, the number of shoots in dodecaploid bamboo was more than double that of seedlings (Figure 3).

**Figure 4:**
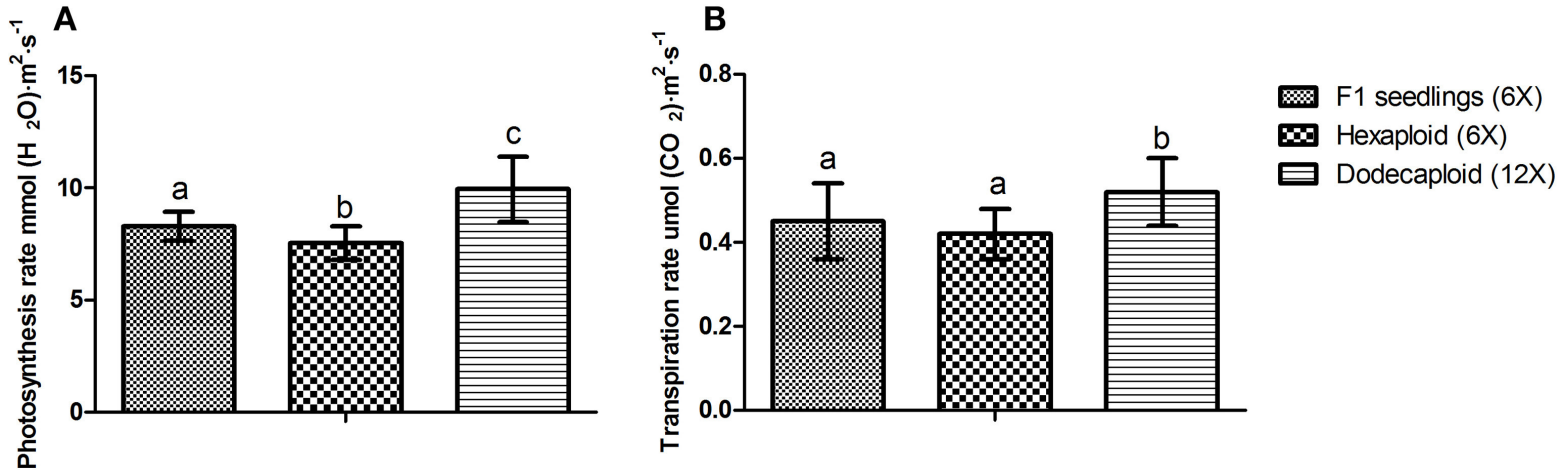
Comparison of photosynthesis and transpiration rates between seedlings and anther-regenerated plants of *Dendrocalamus latiflorus*. **(A)** Photosynthesis rate, **(B)** Transpiration rate. Different capital letters indicate a significant difference between ploidy levels at *p* < 0.05 (Duncan, 1955).

Chlorophyll *a*, chlorophyll *b*, and total chlorophyll levels were previously shown to be significantly higher in dodecaploid lines than in hexaploid lines (Qiao et al., 2013). We found that the photosynthesis and transpiration rates exhibited the same trend, as these values frequently increased with ploidy level in *D. latiflorus* (Figure 4).

### Digital gene expression profiles

To characterize the digital gene expression (DGE) profiles in plants of three different ploidy levels generated from anthers and seedlings grown from seeds, we constructed four DGE libraries and sequenced them using Illumina deep sequencing technology. We calculated the gene expression levels, which are represented by RPKM values, as well as the number of genes belonging to different RPKM intervals (Figure S1). In the triploid (Munro_3) library, the number of genes with RPKM>100 accounted for 40% of expressed genes but only 6% of the total number of genes. Genes with RPKM values between 0 and 10 represented 10% of expressed genes but only approximately 50% of the total number of genes (Figures S1A,B). Genes in hexaploid (Munro_6), dodecaploid (Munro_12), and seedlings (Munro_10) showed similar tendencies. These results indicate that few genes were expressed at high levels, while most were expressed at relatively low levels. Our data reflect the non-uniformity and redundant characteristics of mRNA expression. Figure S2 shows the sequencing saturation levels of the four libraries, revealing that our DGE sequencing covered most of the expressed genes. We tested the randomness of the cDNA fragments by calculating the number of reads matched to different areas of the reference genes (Figure S3). The reads fully covered different loci and were distributed equally, implying that the randomness of the cDNA conformed to the requirements for sequencing and analysis. All of the data were uploaded to an ftp site (ftp.biomarker.com.cn) under the category /zhuory/Munro_DGE (Please contact R. Zhuo for ftp access).

We obtained more than 3.0 million raw tags per library (Table S2). The number of clean tags in each library ranged from 3.03 to 3.34 million. The number of clean tags mapped to transcripts in each library ranged from 2.31 to 2.55 million, accounting for 76.44–76.86% of the total. The number of clean reads that perfectly mapped to transcripts ranged from 1.08 to 1.21 million in different samples, accounting for 45.82–47.36% of the total. Among the four libraries, the number of indel reads ranged from 52,329 to 57,731, whereas the number of indels and mismatched reads ranged from 139,182 to 151,839.

### Analysis of differential gene expression patterns

We performed pair-wise comparisons of the four libraries, implementing a total of six comparisons. We found that 981–3,015 transcripts had significant differences in expression, and a total of 8,396 transcripts were differentially expressed (Table 1). The largest differences in expression occurred between Munro_10 and Munro_6. The smallest difference was observed between Munro_10 and Munro_3, in which only 981 DETs were identified.

**Table 1:** The numbers of DGE transcripts of 6 comparisons between each two samples are shown.

	**Munro_3**	**Munro_6**	**Munro_12**	**Munro_10**
Munro_3		2495	1165	981
Munro_6			2524	3015
Munro_12				1636
Munro_10				

*Munro_3, triploid (3X); Munro_6, hexaploid (6X); Munro_12, dodecaploid (12X); Munro_10, F1 seedlings (6X)*.

### Functional annotation of differentially expressed genes

Several complementary approaches were utilized to annotate the DGE tags. Among the 8396 DETs, 5411, 6497, and 3379 were annotated in the Nr database, Nt database and Swiss-Prot database, respectively. Based on Nr annotation, we carried out Gene Ontology (GO) (Ashburner et al., 2000) analysis. 1118 unigenes were assigned one or more GO terms, including 59.1% in the category molecular functions, 30.1% in biological processes and 10.8% in cellular components (Figure S4). For molecular functions category, Enzyme activity was the most highly represented GO term, followed by binding activity. Genes involved in physiological processes and cellular processes were highly represented in the biological processes category. Cell was the most highly represented category in the cellular components category.

**Figure 5:**
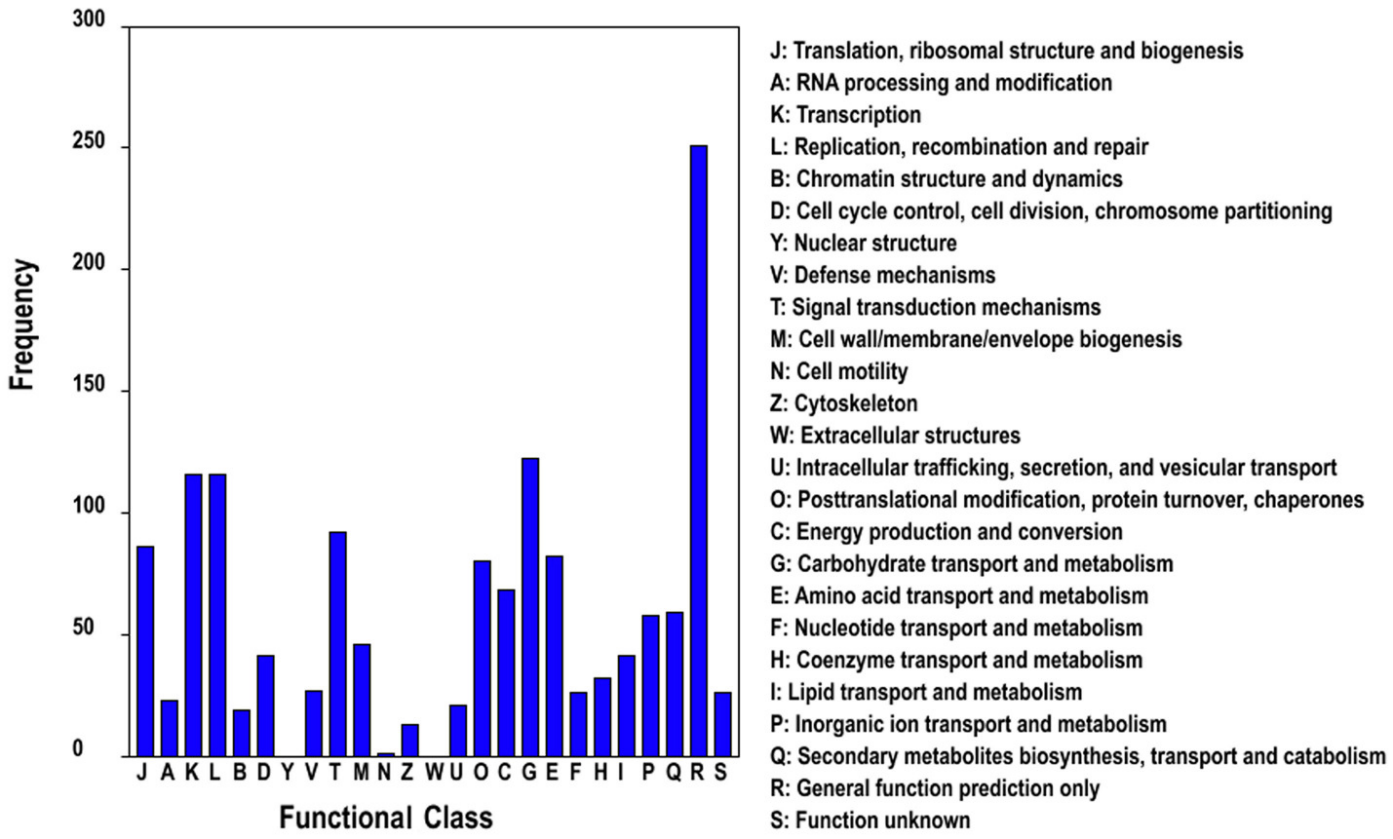
COG classification of differentially expressed genes of different ploidy plants of *Dendrocalamus latiflorus*.

In addition, we subjected all DETs to searches against the Cluster of Orthologous Groups (COG) of proteins database for functional prediction and classification. In total, 1,020 sequences were classified into 25 protein functional families involved in molecular processing, biochemistry metabolism, signal transduction, cellular structure, and so on (Figure 5). The category general function (24.6%) represented the largest group, followed by carbohydrate transport and metabolism (11.7%), replication, recombination and repair (10.9%) and transcription (10.8%).

Annotations of differentially expressed Ma bamboo genes were also subjected to Kyoto Encyclopedia of Genes and Genomes (KEGG) Pathway analysis. A total of 1,103 unigenes were predicted to 128 pathways involved in crucial physiological processes such as photosynthesis, TCA cycle, phenylpropanoid biosynthesis, as well as genetic information processing such as DNA replication, transcription, translation and so on.

**Figure 6:**
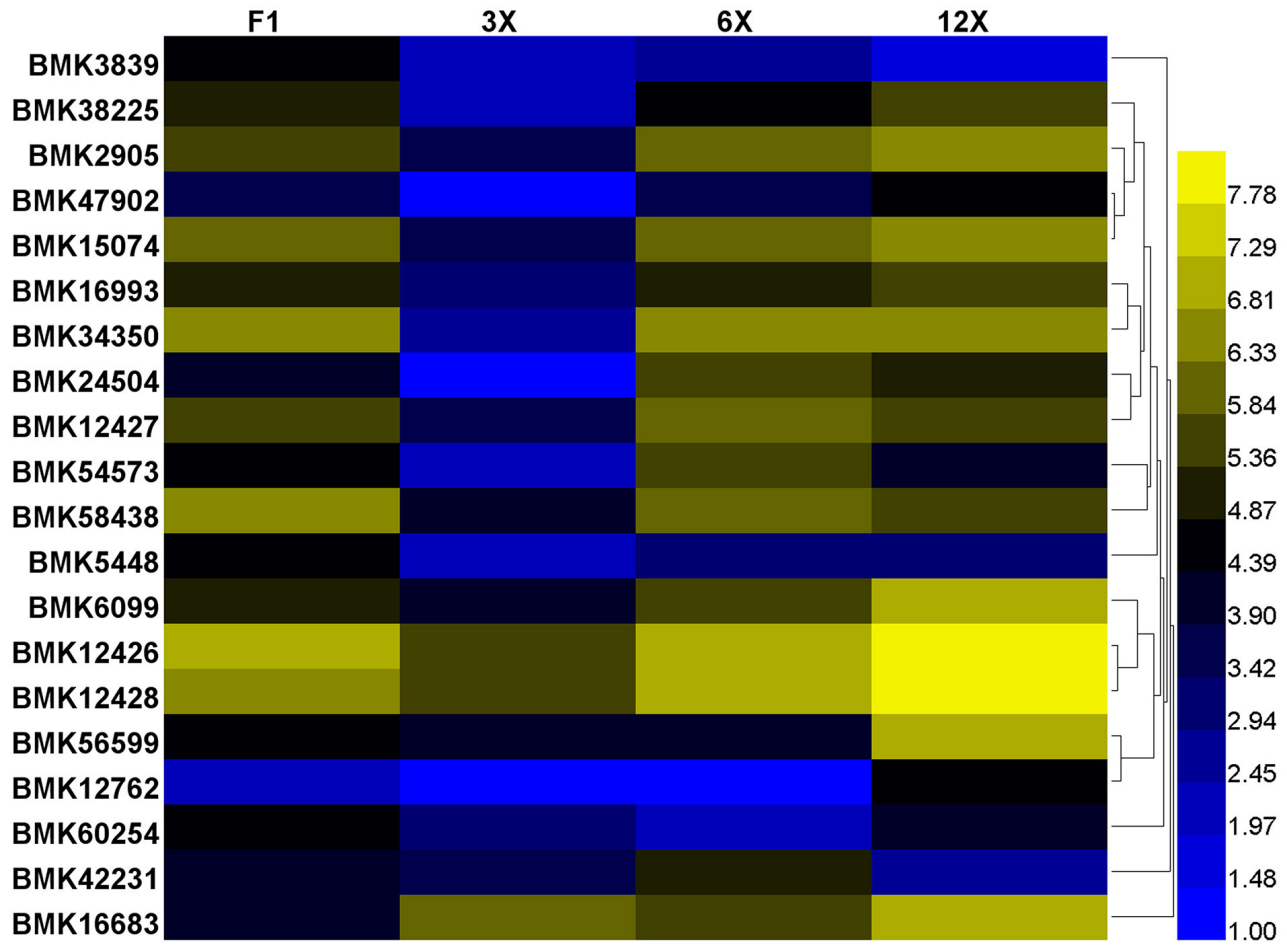
Differential expression of the *expansin* family genes in *Dendrocalamus latiflorus* plants with different ploidy levels based on the DGE data. Color scale represents log2 expression values.

Based on the above annotation results, we are concerned with the DETs involved in regulation of cell proliferation and cell expansion, including transcription regulation, protein biosynthesis and modification, hormonal regulation and cell-wall loosening (Table S3). Among them, we found that most genes of the expansin family were expressed at the highest level in the dodecaploid lines (Figure 6).

### Validation of differentially expressed genes

**Figure 7:**
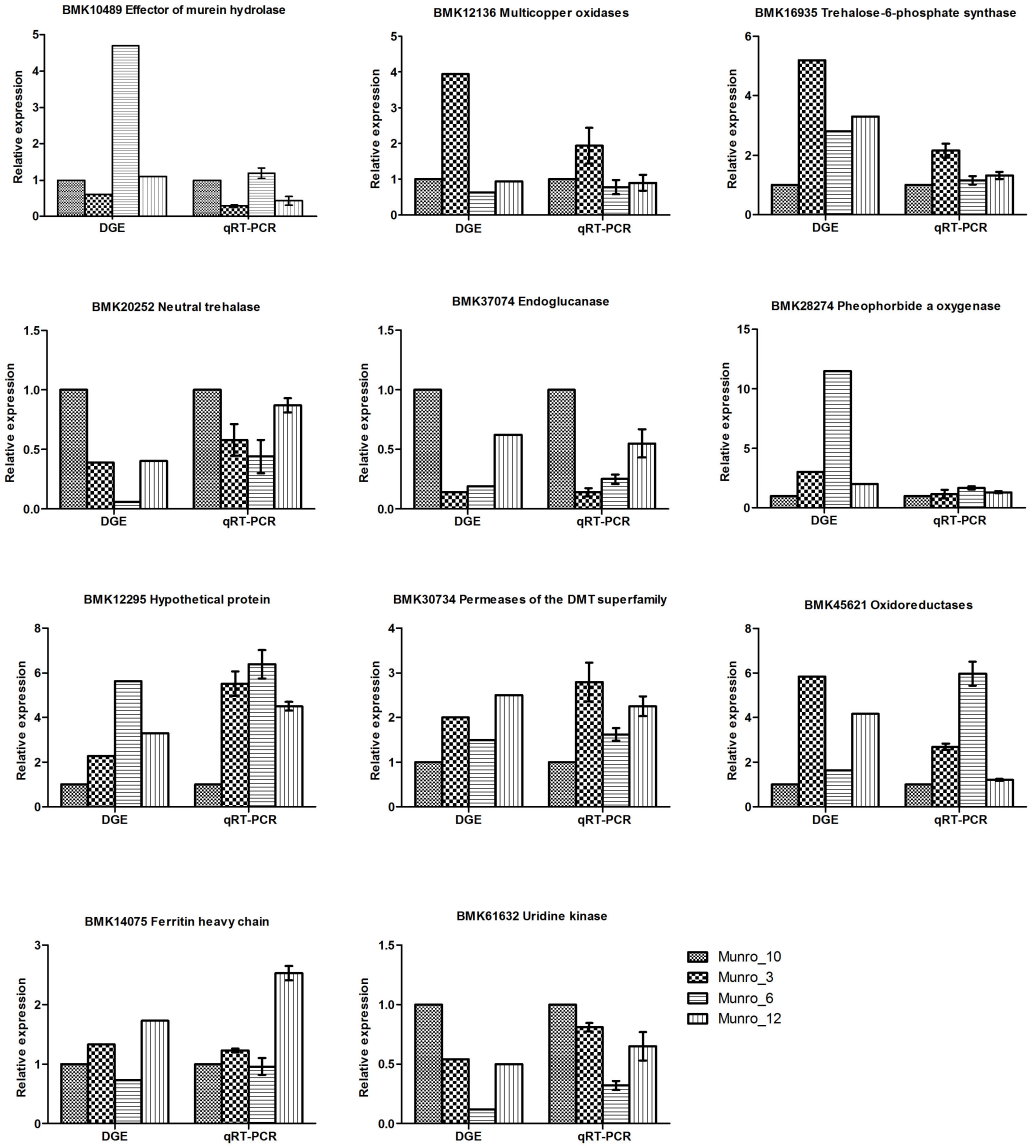
Verification of DGE results by qRT-PCR. Munro_3, triploid (3X); Munro_6, hexaploid (6X); Munro_12, dodecaploid (12X); Munro_10, F1 seedlings (6X).

To validate the results obtained by digital gene expression tag profiling, we chose representative genes differentially expressed from the four libraries and performed quantitative reverse-transcription PCR (qRT-PCR) to assess their characteristics. Three genes, including the genes encoding trehalose-6-phosphate synthase (Eastmond et al., 2002), neutral trehalase (Liu et al., 2011), and endoglucanase (Westereng et al., 2011), which participate in carbohydrate transport and metabolism, were predicted to be dramatically differentially expressed based on DGE analysis. Our qRT-PCR results showed a similar tendency, although the relative ratios of gene expression differed (Figure 7). The expression of *uridine kinase*, which is involved in nucleotide transport and metabolism (Chu et al., 2012), was in accordance with the DGE results. Moreover, the differential expression of *oxidoreductase* genes involved in energy production and conversion, and *laccase-like* genes associated with lignin biosynthesis, was also validated by qRT-PCR. Overall, there was good agreement between the DGE data and the qRT-PCR results.

### Gene co-expression network analysis

To identify coexpression of differentially expressed genes, a network was constructed based on the DGE dataset. Eight gene coexpression modules containing 8,016 unigenes were identified after excluding non- and weakly-expressed genes (Figure S5). These gene modules range in size from 1,969 (turquoise module) to 495 (pink module) genes. Based on this network analysis combined the phenotypic data, we focused on two network hub genes, *expansin B3* (*EXPB3*, BMK12426) and *TCP* (BMK57883), which play prominent roles in cell growth and differentiation. *EXPB*3 was expressed at the highest levels in the dodecaploid lines and at the lowest levels in the triploids. The expression of *TCP* exhibited the opposite trend. In this network, the hub genes *EXPB*3 and *TCP* were coexpressed and directly connected with 184 edges and 53 edges, respectively.

**Figure 8:**
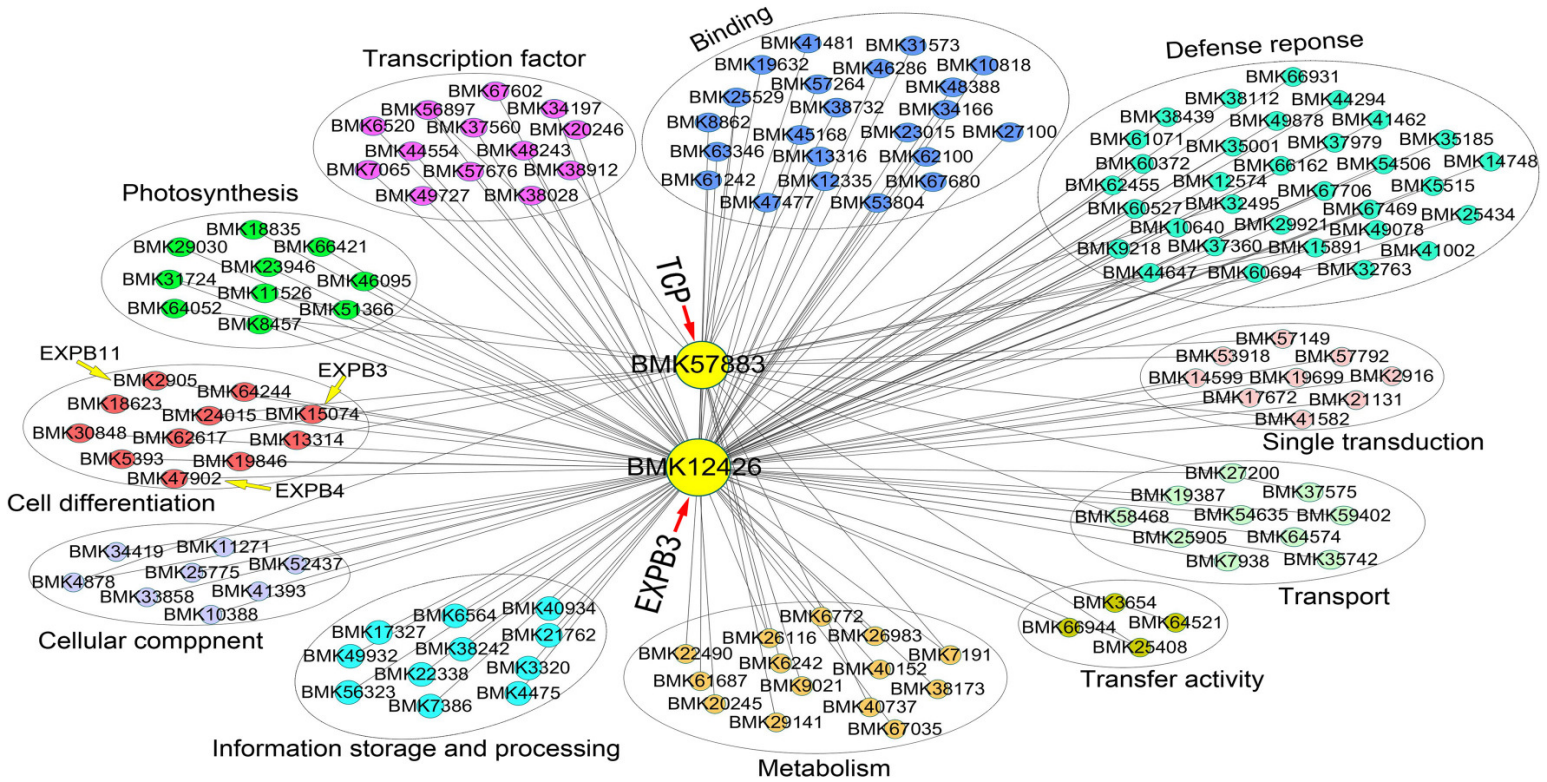
Coexpression network of *EXPB3* and *TCP* based on the DGE data of different ploidy plants of *Dendrocalamus latiflorus*.

We also constructed a subnetwork containing 146 genes by identifying 11 categories related to cell growth, transcription factor, defense response, photosynthesis, and signal transduction, among others (Figure 8). Eleven genes involved in cell growth, such as homologs of β*-EXPANSIN* (BMK15074, BMK2905, BMK47902), *UBC* (*UBIQUITIN-CONJUGATING ENZYME*, BMK18623), and *GIDRP88* (*GROWTH INHIBITION AND DIFFERENTIATION RELATED PROTEIN 88*, BMK24015), may be related to cell wall expansion, cell cycle control, and cell differentiation. Ten photosynthesis-related genes were found in the network, including genes encoding two photosystem II proteins (BMK23946 and BMK31724), chlorophyll a-b binding protein (BMK66421), and glutamyl-tRNA reductase (BMK29030). In addition, 13 and 22 genes belonged to the categories transcription factor and other binding activities, respectively. These genes, such as *myeloblastosis* (*MYB*) superfamily genes (BMK38028 and BMK7065), *Dof* (BMK56897), and *basic-leucine zipper* (*bZIP*) gene *HY5*, regulate many pathways involved in plant growth. We identified 32 genes related to defense responses in the network, which may regulate cellular status to help plants adapt to the environment. Finally, we identified numerous genes with unknown functions in the network (not shown in the figure), perhaps due to the relative lack of gene annotations for bamboo species; these genes represent good candidates for further investigation. Importantly, we can utilize this network to identify novel genes that interact with *EXPB3* and *TCP* in future studies.

## Discussion

In the plant kingdom, polyploidy is remarkably common and polyploidization can be a major driving force for facilitating the plant breeding. Polyploidy has substential phenotypic effects, such as increased cell and organ size, and sometimes greater vigor and biomass, and additional phenotypic and molecular variation can arise shortly after polyploid formation. Compared to diploid parent, doubled diploid citrus rootstock (C. junos cv. Ziyang xiangcheng) has larger and thicker leaves, shorter plant height, bigger stomata and lower stomatal density. Comparative metabolic and transcriptional analysis suggests its potential value for stress resistance improvement (Tan et al., 2015). Cell size and organ thickness were positively correlated with the ploidy level in potato (Stupar et al., 2007). With the increase of genome dosage, the giantism in cells and organs was obvious and the photosynthetic rate was higher (Zhou et al., 2015).

Similarly, we found that the fusoid cells and stomata in leaves of dodecaploids were bigger than those in other ploidies of *D. latiflorus*. Fusoid cells are unique to the grass family and their functions are not clear. Vieira et al. (2002) suggested that fusoid cells may be related to water storage and transportation in the mesophyll cells, based on their position in the leaf. Fusoid cells are colorless and transparent, and therefore facilitate light penetration into the mesophyll cells for photosynthesis (Clayton and Renvoize, 1986). Long et al. speculated that greater photosynthetic ability of 1-year-old bamboo may be associated with more developed fusoid cells of the leaves (Chunling et al., 2015). Consistent with this idea, our results showed that dodecaploid bamboo had a higher photosynthetic rate compared to other ploidies. The dodecaploid lines also showed the highest transpiration rate, which might be related to the enlargement of stomatal size. Shoot number is one of the most important economic traits of bamboo. The effect of increased ploidy on cell differentiation might also lead to the increases in epidermis, leaf thickness and shoot number. The capacity for photosynthesis and stress resistance in *Phyllostachys edulis* “Pachyloen” with thicker cuticle and epidermis cells was higher than that in *Ph. Edulis* (Chunling et al., 2015). Therefore, we speculate that stress resistance may also be enhanced with the increase of ploidy level in *D. latiflorus*, an idea that awaits further study.

To understand the mechanisms underlying the phenotypic differences in bamboo plants with different ploidy levels, the genome-wide gene expression profiles of four bamboo lines were determined by “second-generation” sequencing of short cDNA tags. We found that 981–3,015 transcripts had significant differences in expression and that the total number of differentially expressed genes was 8,396. These differentially expressed genes, which are predicted to be potentially involved in plant growth and development, merit further investigation.

A large number of genes involved in regulation of cell proliferation and/or cell expansion have been identified, and their up- or down-regulated expression change cell size and accelerate plant growth by means of transcription regulation, protein biosynthesis and modification, hormonal regulation and cell-wall loosening, and so on Huang et al. (2013). Here, we found these genes were also differentially expressed in different ploidies of Ma bamboo. Previous studies have reported correlation of cell wall modification, plant growth rate and phenotype changes with regulation of *Expansin* gene expression in rice (Lee and Choi, 2005; Ma et al., 2013), tobacco (Li et al., 2013; Xu et al., 2014) and *Arabidopsis* (Hu et al., 2013; Goh et al., 2014). Most reported findings support the role of expansin as a major protein to induce cell wall extension, larger cells, taller plants and longer roots. The data strongly suggest the quantitative importance of polyploidy-associated cell expansion as a determinant of fruit weight in tomato (Cheniclet et al., 2005). In addition, the TCP domain protein TEOSINTE BRANCHED1 (TB1) is a putative transcriptional regulator that represses bud outgrowth in grasses (Takeda et al., 2003). Two *TB1* homologs, *BRANCHED1* (*BRC1, AtTCP18*) and *BRANCHED*2 (*BRC2, AtTCP12*), are expressed in axillary buds, and mutants with reduced activity of either gene show increased branching (Finlayson, 2007). The orthologs *SlBRC1a* and *SlBRC1b* have similar functions in axillary bud initiation and outgrowth in tomato. Two genes (BMK57883 and BMK59438) encoding TCP domain proteins were expressed at their lowest levels in dodecaploid bamboo and whether they are related to the shoots number should be further verified. Importantly, our coexpression network had *EXPB3* and *TCP* as hub genes that were coexpressed with 222 genes.

## Conclusion

In conclusion, our results indicate that differences in characteristics are associated with differences in ploidy level in *D. latiflorus* plants. Transcriptome profiling and sequencing were performed and 8,396 differentially expressed genes were revealed in different ploidy bamboos. These differentially expressed genes especially in cell growth and differentiation, which are predicted to be potentially involved in plant growth and development, needed further experimental verification. Additionally, two candidate genes, *EXPB3* and *TCP* with potenitial regulatory roles in cell division and differentiation, were identified through gene coexpresseion network analysis. These results highlight the significance of potential applications of polyploidy, and provide valuable information for the genetic breeding of bamboo species.

## Author contributions

RZ designed the study. KS, HL, HY, and YY performed the experiments. GQ and ML analyzed the data and wrote the paper. All authors read and approved the manuscript.

### Conflict of interest statement

The authors declare that the research was conducted in the absence of any commercial or financial relationships that could be construed as a potential conflict of interest.
