# Supplementary material for: Phenotypic and Comparative Transcriptome Analysis of Different Ploidy Plants in Dendrocalamus latiflorus Munro
Source: Front Plant Sci. 2017 Aug 8;8:1371. doi: 10.3389/fpls.2017.01371 (PMC5550759; doi:10.3389/fpls.2017.01371)
Supplement: Supplementary file 1 [file Image1.PDF]

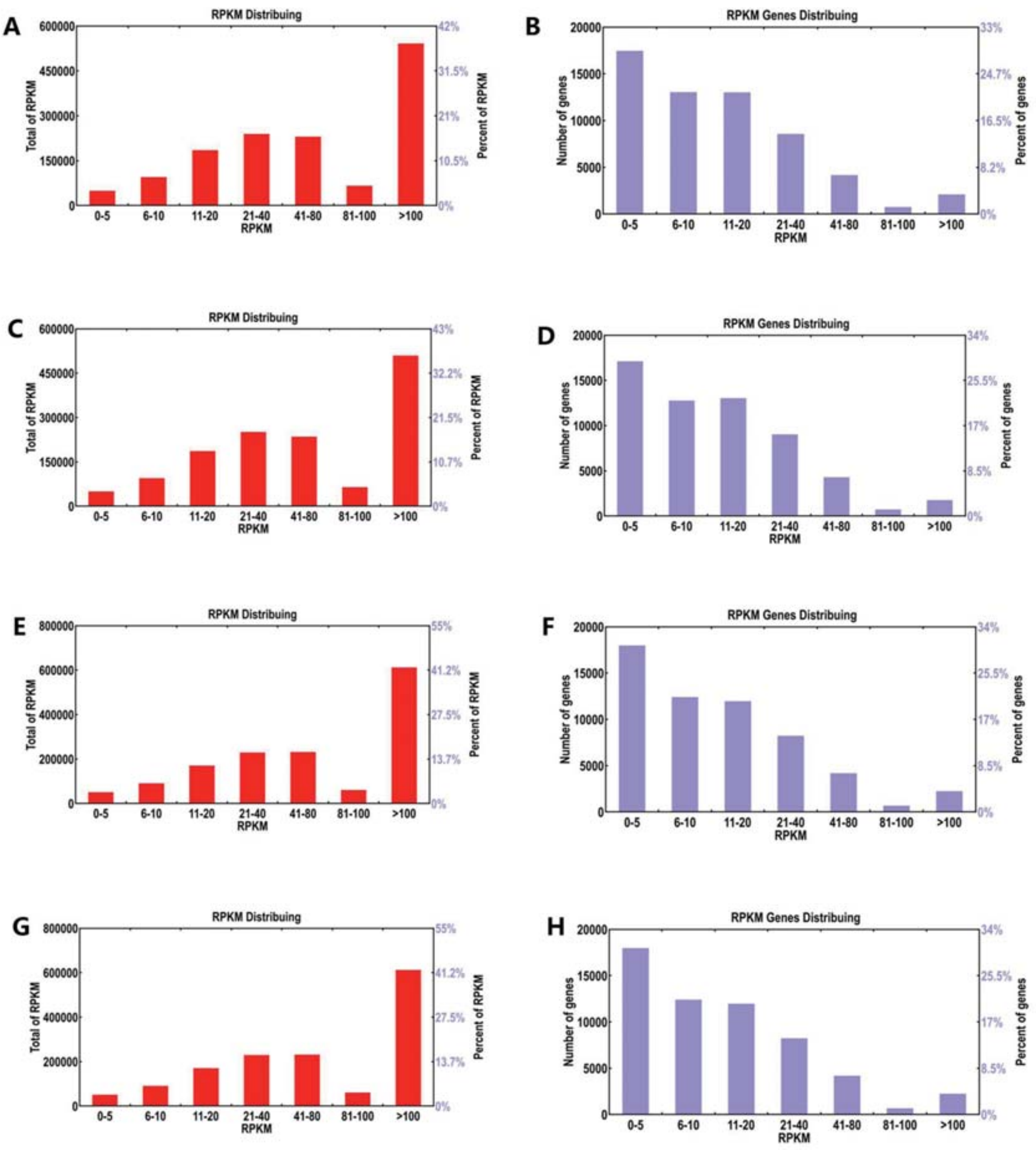

Figure S1 RPKM distribution (left) and the number of genes belonging to different RPKM intervals (right) of four libraries of *Dendrocalamus latiflorus*. A and B, triploid (3X), C and D, hexaploid (6X), E and F, dodecaploid (12X), G and F, F1 seedlings (6X).
