# Supplementary material for: Phenotypic and Comparative Transcriptome Analysis of Different Ploidy Plants in Dendrocalamus latiflorus Munro
Source: Front Plant Sci. 2017 Aug 8;8:1371. doi: 10.3389/fpls.2017.01371 (PMC5550759; doi:10.3389/fpls.2017.01371)
Supplement: Supplementary file 3 [file Image3.PDF]

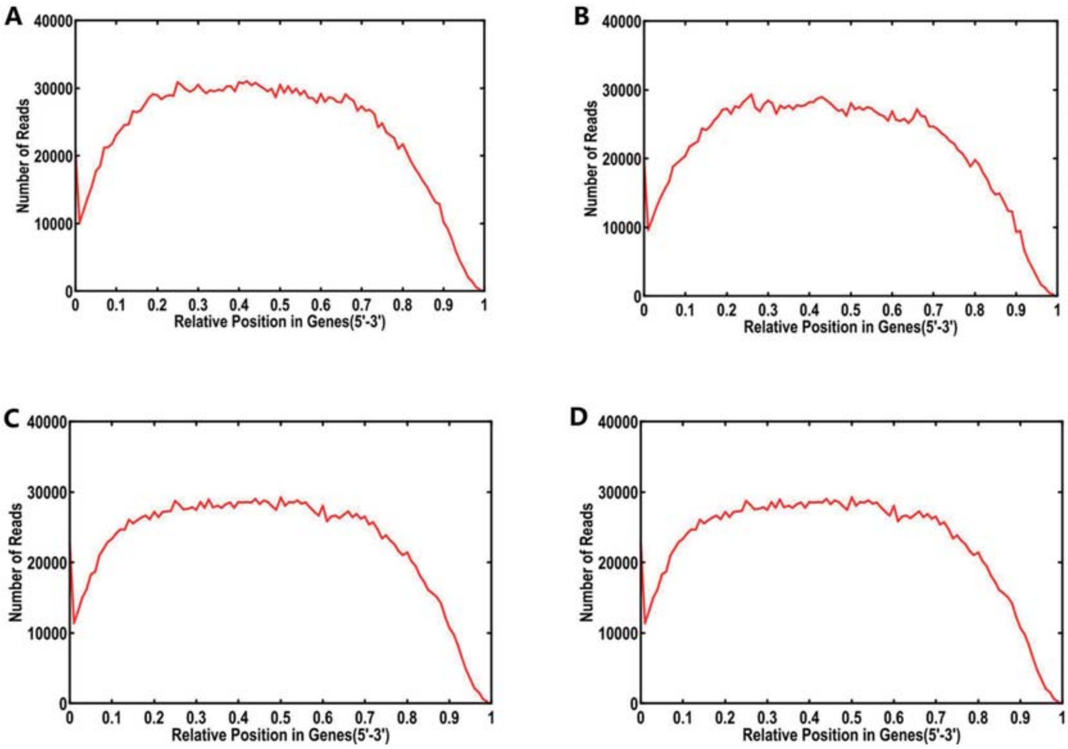

Figure S3 cDNA fragment randomness of the four libraries of *Dendrocalamus latiflorus*. A, triploid (3X), B, hexaploid (6X), C, dodecaploid (12X), D, F1 seedlings (6X).
