# Supplementary material for: Phenotypic and Comparative Transcriptome Analysis of Different Ploidy Plants in Dendrocalamus latiflorus Munro
Source: Front Plant Sci. 2017 Aug 8;8:1371. doi: 10.3389/fpls.2017.01371 (PMC5550759; doi:10.3389/fpls.2017.01371)
Supplement: Supplementary file 4 [file Image4.PDF]

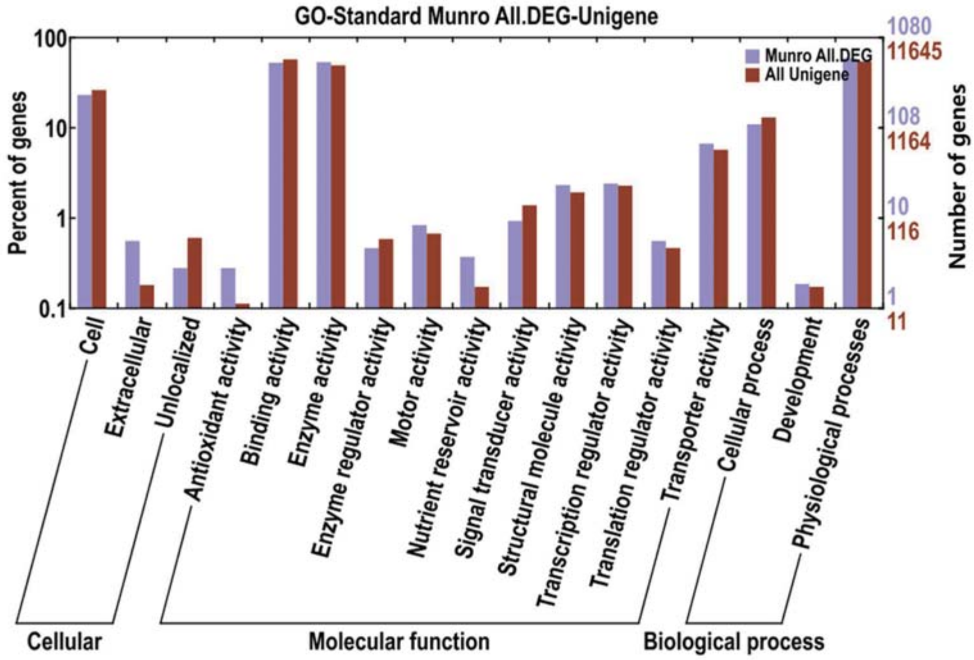

Figure S4 Functional annotation of differentially expressed genes of different ploidy *Dendrocalamus latiflorus* based on gene ontology (GO) categorization.
