# Supplementary material for: Phenotypic and Comparative Transcriptome Analysis of Different Ploidy Plants in Dendrocalamus latiflorus Munro
Source: Front Plant Sci. 2017 Aug 8;8:1371. doi: 10.3389/fpls.2017.01371 (PMC5550759; doi:10.3389/fpls.2017.01371)
Supplement: Supplementary file 5 [file Image5.PDF]

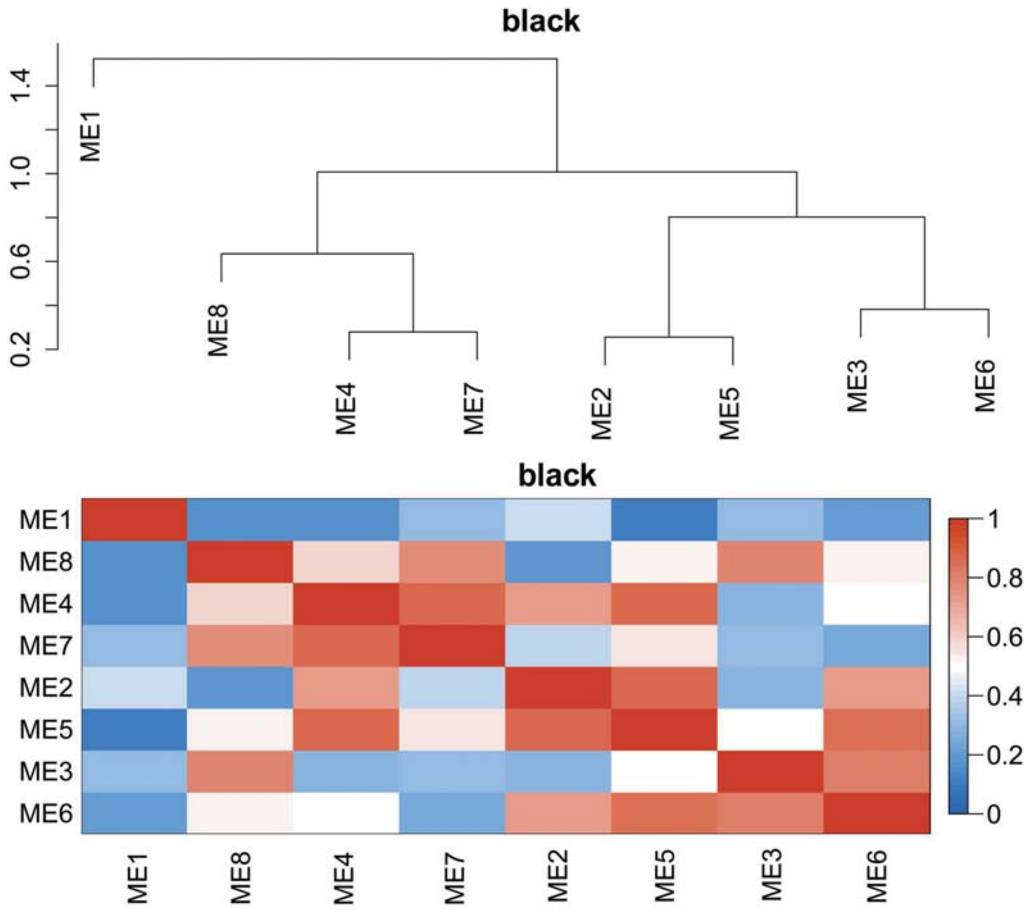

Figure S5 WGCNA coexpression modules based on DEG data of F1 seedlings and anther-regenerated plants with three ploidy levels of *Dendrocalamus latiflorus*. Different colors represented different modules of coexpressed genes.
