# Supplementary material for: Phenotypic and Comparative Transcriptome Analysis of Different Ploidy Plants in Dendrocalamus latiflorus Munro
Source: Front Plant Sci. 2017 Aug 8;8:1371. doi: 10.3389/fpls.2017.01371 (PMC5550759; doi:10.3389/fpls.2017.01371)
Supplement: Supplementary file 6 [file Table1.PDF]

Table S1 Primer sequences designed for differential expressed genes identified by DGE analysis

Gene	Primer sequences	Annotation	Function
**BMK_10489**	F:5'-CAATACAGTGGAAGCGAGCA-3'<br>R:5'-CCATGAACAAACCGTCTCCT-3'	effector of murein hydrolase	Cell wall/membrane/ envelope biogenesis
**BMK_12136**	F:5'-GGAAGGTGACGTTGTTGTTG-3'<br>R:5'-TCAATGTGACAATGGCGACT-3'	laccase-like	Secondary metabolites biosynthesis, transport and catabolism
**BMK_16935**	F:5'-TCGCAGCCTTTTCTCAGATT-3'<br>R:5'-CTCCCATCACACAAGCACAC-3'	Trehalose-6-phosp hate synthase	Carbohydrate transport and metabolism
**BMK_20252**	F:5'-CGCTATGTCCCGTTCCATCTTG-3'<br>R:5'-GGCCAGAAAGTGCAACAATCG-3'	Neutral trehalase	Carbohydrate transport and metabolism
**BMK_37074**	F:5'-AAGCCAGAGGAGCTGCCAAA-3'<br>R:5'-AGTCCTCGGTCGCGTAAATCA-3'	Endoglucanase	Carbohydrate transport and metabolism
**BMK_61632**	F:5'-AAGAGCCCCGTGCCATTGTT-3'<br>R:5'-TGGAGATGGACGGGCAATTC-3'	Uridine kinase	Nucleotide transport and metabolism
**BMK_12295**	F:5'-TGCCTTCGTGAAGGTGAGCA-3'<br>R:5'-TGAACCATTCACCGGATCAGC-3'	Auxin-responsive protein IAA13	regulation of transcription
**BMK_30734**	F:5'-GCCGCAAAGATCATCGGAAC-3'<br>R:5'-TGCAGCAGAAGCCAAAAGGA-3'	Permeases of the DMTsuperfamily	Cellular Component
**BMK_45621**	F:5'-GGGAGATTCTTGCGGAAATCGT-3'<br>R:5'-CTGAAGCATCGGCGTCAACA-3'	oxidoreductases	Energy production and conversion
**BMK_14075**	F:5'-TACTTTCGGCTGCGCCATGT-3'<br>R:5'-CGGGATGCTGCTGAAGGAAAT-3'	ferritin heavy chain	Porphyrin and chlorophyll metabolism
**BMK_28274**	F:5'-GCGACCTCGTCATCTGGAA-3'<br>R:5'-CCTGAGCCATCGAATGACC-3'	pheophorbide a oxygenase	oxidoreductase activity
