# Supplementary material for: Phenotypic and Comparative Transcriptome Analysis of Different Ploidy Plants in Dendrocalamus latiflorus Munro
Source: Front Plant Sci. 2017 Aug 8;8:1371. doi: 10.3389/fpls.2017.01371 (PMC5550759; doi:10.3389/fpls.2017.01371)
Supplement: Supplementary file 7 [file Table2.PDF]

Table S2 Statistics of DGE library sequencing and tag mapping.

**Summary**	**Munro_3**	**Munro_6**	**Munro_12**	**Munro_10**
Clean Tags	3,283,402	3,028,221	3,078,729	3,337,293
GC(%)	49.98	49.54	49.89	48.14
CycleQ20%	100	100	100	100
Mapped Reads	2,516,055	2,314,741	2,365,883	2,552,705
Mapped Reads (%)	76.65	76.44	76.85	76.49
Perfect Mapped Reads	1,181,575	1,090,456	1,084,116	1,209,012
Perfect Mapped Reads (%)	46.96	47.11	45.82	47.36
Mismatch Reads	1,124,928	1,029,737	1,077,766	1,139,904
Mismatch Reads (%)	44.71	44.49	45.55	44.65
Indel Reads	57,713	55,366	52,329	55,721
Indel Reads (%)	2.29	2.39	2.21	2.18
Indel and Mismatch Reads	151,839	139,182	151,672	148,068
Indel and Mismatch Reads (%)	6.03	6.01	6.48	5.80
Identities	0.98161995	0.981785498	0.981137035	0.981652757
